# Detection of Extracochlear Electrodes in Cochlear Implants with Electric Field Imaging/Transimpedance Measurements: A Human Cadaver Study

**DOI:** 10.1097/AUD.0000000000000837

**Published:** 2020-01-07

**Authors:** Simone R. de Rijk, Yu C. Tam, Robert P. Carlyon, Manohar L. Bance

**Affiliations:** 1 Department of Clinical Neurosciences, University of Cambridge, Cambridge, United Kingdom; 2 Emmeline Centre, Cambridge University Hospitals NHS Foundation Trust, Cambridge, United Kingdom; 3 MRC Cognition and Brain Sciences Unit, University of Cambridge, Cambridge, United Kingdom.

**Keywords:** Cochlear Implants, Extracochlear Electrodes, Electric Field Imaging, SCINSEVs, Transimpedance measurements

## Abstract

**Objectives:**

Extracochlear electrodes in cochlear implants (CI), defined as individual electrodes on the electrode array located outside of the cochlea, are not a rare phenomenon. The presence of extracochlear electrodes frequently goes unnoticed and could result in them being assigned stimulation frequencies that are either not delivered to, or stimulating neurons that overlap with intracochlear electrodes, potentially reducing performance. The current gold-standard for detection of extracochlear electrodes is computed tomography (CT), which is time-intensive, costly and involves radiation. It is hypothesized that a collection of Stimulation-Current-Induced Non-Stimulating Electrode Voltage recordings (SCINSEVs), commonly referred to as “transimpedance measurements (TIMs)” or electric field imaging (EFI), could be utilized to detect extracochlear electrodes even when contact impedances are low. An automated analysis tool is introduced for detection and quantification of extracochlear electrodes.

**Design:**

Eight fresh-frozen human cadaveric heads were implanted with the Advanced Bionics HiRes90K with a HiFocus 1J lateral-wall electrode. The cochlea was flushed with 1.0% saline through the lateral semicircular canal. Contact impedances and SCINSEVs were recorded for complete insertion and for 1 to 5 extracochlear electrodes. Measured conditions included: air in the middle ear (to simulate electrodes situated in the middle ear), 1.0% saline in the middle ear (to simulate intraoperative conditions with saline or blood in the middle ear), and soft tissue (temporal muscle) wrapped around the extracochlear electrodes (to simulate postoperative soft-tissue encapsulation of the electrodes). Intraoperative SCINSEVs from patients were collected, for clinical purposes during slow insertion of the electrode array, as well as from a patient postoperatively with known extracochlear electrodes.

**Results:**

Full insertion of the cochlear implant in the fresh-frozen human cadaveric heads with a flushed cochlea resulted in contact impedances in the range of 6.06 ± 2.99 kΩ (mean ± 2SD). Contact impedances were high when the extracochlear electrodes were located in air, but remained similar to intracochlear contact impedances when in saline or soft tissue. SCINSEVs showed a change in shape for the extracochlear electrodes in air, saline, and soft tissue. The automated analysis tool showed a specificity and sensitivity of 100% for detection of two or more extracochlear electrodes in saline and soft tissue. The quantification of two or more extracochlear electrodes was correct for 84% and 81% of the saline and soft tissue measurements, respectively.

**Conclusions:**

Our analysis of SCINSEVs (specifically the EFIs from this manufacturer) shows good potential as a detection tool for extracochlear electrodes, even when contact impedances remain similar to intracochlear values. SCINSEVs could potentially replace CT in the initial screening for extracochlear electrodes. Detecting migration of the electrode array during the final stages of surgery could potentially prevent re-insertion surgery for some CI users. The automated detection tool could assist in detection and quantification of two or more extracochlear electrodes.

## INTRODUCTION

Cochlear implants (CIs) are electronic devices that convert sounds to direct intracochlear stimulation of the auditory nerve. For many patients with severe to profound hearing loss, cochlear implantation is a life-transforming technology (Gaylor et al. 2013). However, a substantial subset of CI users performs poorly (Bodmer et al. 2007; Gifford et al. 2008; Holden et al. 2013). Understanding the reason for this poor performance is of great clinical relevance (Pisoni et al. 2017).

One finding associated with poor performance is the presence of extracochlear electrodes (Hilly et al. 2016; Rivas et al. 2008), defined as individual electrodes on the electrode array that are located outside of the cochlea due to incomplete insertion or extrusion. The prevalence of extracochlear electrodes is estimated to be between 9.2% and 13.4% (Coombs et al. 2014; Holder et al. 2018). The mechanism behind extracochlear electrodes is commonly categorized into incomplete insertion, for example, due to ossification or cochlear malformations, or electrode migration post-implantation (Holder et al. 2018). In case of incomplete insertion, the surgeon is aware of the extracochlear electrodes and the auditory map can be adjusted accordingly. In case of post-implantation electrode migration, these extracochlear electrodes are not always detected, particularly with standard telemetry. A recent retrospective study by Holder et al. found that 60% of the CI recipients with extracochlear electrodes, as identified by computed tomography (CT), were not identified by any audiology measures such as contact impedances, evoked compound action potentials (ECAPs) or auditory mapping. Only 6% of the cases were identified during the cochlear implantation itself.

Post-implantation migration is the second most common reason for revision cochlear implantation, emphasizing the clinical issues related to electrode migration (Brown et al. 2009; Connell et al. 2008; Green et al. 2004; Wang et al. 2014). Prevalence of electrode migration varies from 7.4% to 29%, and seems to occur more frequently and to a greater extent in lateral-wall electrodes than perimodiolar electrodes (Mittmann et al. 2015; van der Marel et al. 2012). It is suggested that these migrations mostly happen during perioperative conditions, for example, while closing the skin when minor manipulations of the implant might occur, or in the first few weeks postoperative when the electrode array is not yet immobilized by ossification or fibrous tissue (van der Marel et al. 2012). Some surgeons attempt to fix the electrode to prevent migration with either soft tissue packing, recessing electrodes into notches in the facial recess, or using bone-wax.

Although most CI users do not seem to experience any symptoms with small migrations of the electrode array, migrations leading to the presence of extracochlear electrodes are related to a decrease in performance and/or non-auditory sensations and are associated with elevated aided detection thresholds (Dietz et al. 2016; Hilly et al. 2016; van der Marel et al. 2012; Rivas et al. 2008; Smullen et al. 2005). We hypothesize that there are generally four ways in which the presence of extracochlear electrodes may lead to poorer performance: (1) the extracochlear electrodes might not adequately stimulate the cochlear nerve. This is particularly troublesome if they are unrecognized and assigned frequencies in the map, as they essentially become “parasitic” in the frequency map. Other causes include that (2), the extracochlear electrodes might stimulate the same neural region as the most basal intracochlear electrode leading to a possible loss of frequency selectivity and/or (3) lead to a possible loss of stimulation in the apical region due to decreased insertion depth. (4) Additionally, incorrect pitch placement of the electrode array in the cochlea due to partial insertion could cause the recipient to experience a pitch shift, which may delay recipients in adapting to the sound from the CI at switch on.

The current gold standard for detection of extracochlear electrodes or electrode migration is CT. However, this is not part of the current standard postoperative clinical care for CI users in all centers. Furthermore, in many countries, CT scans have to be separately scheduled, are time-intensive, costly, and involve radiation, which is particularly worrisome in children because of the long-term neoplasia induction risks (Brenner & Hall 2007). Other clinical measures such as ECAPs and auditory mapping seem insufficient for detection of extracochlear electrodes, as an ECAP response and auditory response can be present in case of extracochlear electrodes, possibly stimulating the same tonotopic region as the most basal intracochlear electrode (Holder et al. 2018). Although it has been reported that basal contact impedances increase over time when electrodes migrate outside of the cochlea (Dietz et al. 2016), contact impedances are in the normal range for the majority of these CI users (Coombs et al. 2014; Holder et al. 2018).

**Fig. 1.**
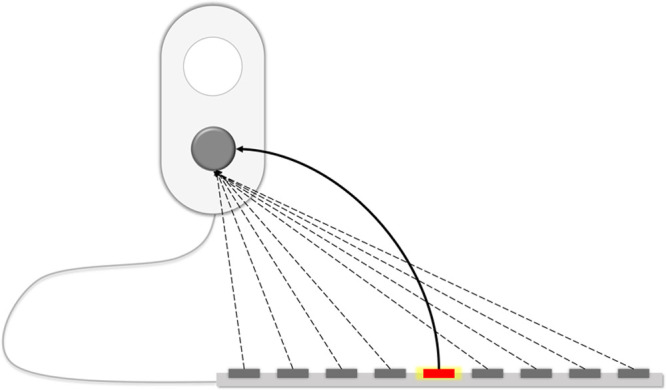
Schematic representation of stimulation-current-induced non-stimulating electrode voltage recordings (SCINSEVs) and contact impedances. An individual electrode on the electrode array is stimulated with current (red electrode on the middle of the array) and voltage is measured between this stimulating electrode and case ground (solid arrow). This measure, normalized by the input current, is referred to as the contact impedance. SCINSEV recordings are made by measuring the voltage between the non-stimulated electrodes and the case ground electrode (dashed arrows), normalized by the input current, when stimulating 1 electrode (red electrode on the middle of the array in this example).

The aim of this study was to investigate whether a collection of Stimulation-Current-Induced Non-Stimulating Electrode Voltage recordings (SCINSEVs), commonly referred to as “transimpedance measurements,” could be utilized to detect extracochlear electrodes in CIs. SCINSEVs are measurements of the voltage passively induced on non-stimulated electrodes, with reference to the ground electrode, when other electrodes on the electrode array are stimulated in turn (Fig. 1). SCINSEVs differ from the routinely measured contact impedances, as contact impedances are recordings of the voltage at the same electrode that is being stimulated. Furthermore, because current is flowing through this electrode to the ground electrode, contact impedances are much affected by the electrode-electrolyte interface.

**Fig. 2.**
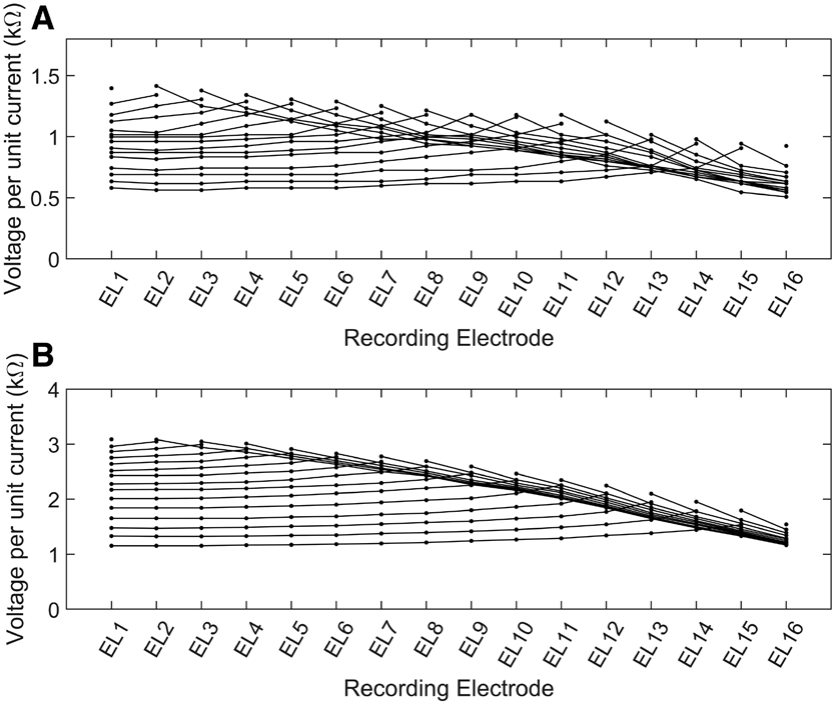
SCINSEVs with Advanced Bionics implants. EL1 (electrode 1) refers to the most apical electrode, while EL16 (electrode 16) refers to the most basal electrode. A, Intraoperative SCINSEV recording in a live patient. B, SCINSEV recording in a cadaver after flushing the cochlea with 1.0% saline.

The use of SCINSEV recordings is attractive as it is built into the testing and fitting or research software of most CI companies, and only takes minutes to perform. For example, SCINSEV recordings can easily be performed in the final stages of cochlear implantation surgery or in the outpatient clinic setting. These types of recordings have been labeled differently in the various companies’ clinical research software. Cochlear Corporation (Cochlear Ltd., Sydney, Australia) refers to these measurements as Transimpedance Measurements (TIMs). Advanced Bionics (Advanced Bionics LLC, Valencia, CA) refers to these SCINSEV measurements as Electric Field Imaging (EFI) (Vanpoucke et al. 2004). Therefore, we chose to use a simple descriptor of the measurements, SCINSEVs, rather than a company-specific term, which also highlights that these are induced voltage recordings on an electrode distant to the one being stimulated, and not impedance recordings, as reported in the clinical software. SCINSEVs provide information on the spatial distribution of current-induced voltage along the longitudinal axis, by measuring all possible combinations of stimulating and recording electrodes (Fig. 1). SCINSEV measurements by Advanced Bionics, referred to as EFIs, have previously been used to identify current shunts, predict loudness, and detect tip fold-overs (Berenstein et al. 2010; Vanpoucke et al. 2004; Zuniga et al. 2017). Although contact impedances (Fig. 1) could potentially be used as a marker for extracochlear electrodes surrounded by air, it is unlikely that they are elevated when these extracochlear electrodes are surrounded by fluids, for example, saline or blood at the end of surgery, or if the extracochlear electrodes are covered by soft tissue, for example, fibrotic tissue or soft tissue packing of the round window. We hypothesized that, even when contact impedances are low on the extracochlear electrodes, a difference might be seen in the spread along the intracochlear versus extracochlear electrodes when measuring SCINSEVs. A second aim of our work was to explore whether the number of electrodes outside of the cochlea could be quantified using SCINSEVs. A third aim was to investigate whether we could automatize the identification and quantification process of extracochlear electrodes, as identified by SCINSEVs, so that clinicians unfamiliar with SCINSEV measurements could still benefit from it as a detection tool.

## MATERIALS AND METHODS

### Human Cadaveric Tissue

Fresh-frozen human cadaveric heads were procured from the Anatomy Gifts Registry (USA) for surgical training and research within a longstanding surgical training facility in our institution. The conduct of this study was approved by our institutional Human Biology Research Ethics Committee (Project no. HBREC.2018.25)

### Implantation in Human Cadaveric Heads

Eight fresh-frozen human cadaveric heads were implanted with the same Advanced Bionics (AB) (Advanced Bionics LLC, Valencia, CA) HiRes90K receiver stimulator with a HiFocus 1J lateral-wall electrode. This electrode array has 16 electrodes and is numbered in such a way that electrode 1 corresponds to the most apical electrode, while electrode 16 corresponds to the most basal electrode. A mastoidectomy, posterior tympanotomy and an extended round window approach were performed to gain access to the round window. As in clinical implantation, the case ground electrode was located underneath the temporal muscle. To remove air or debris present inside the cochlea while still maintaining the conductive milieu of perilymph, the cochlea was flushed with saline. An opening was made in the lateral semi-circular canal (LSCC), using a 1-mm diamond burr, and the cochlea was flushed through this artificial opening in the LSCC until clear saline exited the round window opening. The incus was removed from the middle ear to gain better visual access to the electrode array. A saline concentration of 1.0% (GIBCO distilled water, sodium chloride S/3160/60, Fisher Scientific, MA) was used to flush the cochlea, to approximate the conductivity of perilymph at 1.79 S/m (Baumann et al. 1997). The opening in the LSCC was closed with non-conductive material (Blu Tack) after flushing. Resulting SCINSEV recordings were similar in shape to intraoperative SCINSEV recordings in live CI users, while the range of values in intraoperative recordings differs from the range in cadaver recordings, as can be seen in the example in Figure 2.

### SCINSEV Recordings

The implant was driven by the external sound processor (AB Clarion Platinum), connected to a Clinician’s Programming Interface (CPI-2, AB-6500 Clarion) and a computer. Contact impedances and the company-specific SCINSEVs (EFIs) were measured using AB’s Volta software (version 1.1.1.21032). Volta measures the SCINSEVs by presenting a cathodic-anodic biphasic pulse with an amplitude of 32 µA and phase duration of 36 µsec and presents the measured voltage per unit of current injected at the electrode (voltage per unit current expressed in kiloohm).

### Experimental Design

SCINSEVs and contact impedances were measured at full insertion and with a systematically varying number of 1 to 5 extracochlear electrodes. The actual number of extracochlear electrodes was determined by inspection through a surgical microscope. Extracochlear electrode SCINSEV measurements were made with: (1) air in the middle ear, (2) 1.0% saline in the middle ear, and (3) soft tissue (temporal muscle) wrapped around the extracochlear electrodes. Saline was used to simulate the conditions in the middle ear during surgery and directly post-implantation, while soft tissue was used to simulate longer term postoperative conditions when fibrous tissue might be located on the extracochlear electrodes. Each measurement was repeated three times and the mean and standard deviation was taken of these measurements.

### Detection and Quantification of Extracochlear Electrodes

**Fig. 3.**
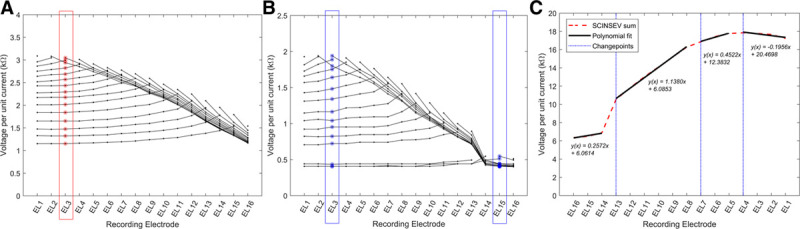
Methods for automatizing the detection and quantification of extracochlear electrodes. EL1 (electrode 1) refers to the most apical electrode, while EL16 (electrode 16) refers to the most basal electrode. A, Example of the calculation of the SCINSEV sum. A SCINSEV for a full insertion in a fresh-frozen cadaveric head is shown. The red markers show the datapoints as collected from recording electrode 3, while stimulating all the other electrodes, that is, 1–2 and 4–16 in this example. In the calculation of the SCINSEV sum, the value of all these datapoints (voltages induced at electrode 3 by stimulation of all other electrodes) are summed to form the SCINSEV sum for that electrode. This is repeated for each recording electrode. B, Example of the calculation of the SCINSEV sum 3 extracochlear electrodes in 1.0% saline in a fresh-frozen human cadaveric head. The blue markers show the datapoints as collected from recording electrode 3 and 15. In the calculation of the SCINSEV sum, the value of all these datapoints (voltages induced at electrode 3 and 15 by stimulation of all other electrodes) are summed to form the SCINSEV sum for that electrode. This is repeated for each recording electrode. C, Example of polynomial fit of the SCINSEV sum for 3 extracochlear electrodes. The sum of a SCINSEV (equal to Figure 4B) is flipped, shown here as a red dashed line. The changepoints are calculated using the *findchangepts* function (MATLAB (vR2018A, MathWorks Inc., Massachusetts, USA). The four segments are fitted to a polynomial, displayed with the corresponding polynomial function.

To automate the detection and quantification process, the SCINSEV data were analyzed as follows. First, the mean of the three measured SCINSEVs was taken. Second, the sum of all recorded points was calculated, so that each recording electrode was represented by one value (as shown in Fig. 3A, with all electrodes intracochlear, and Fig. 3B, with three electrodes extracochlear). Third, MATLAB’s (vR2018A, MathWorks Inc., MA) *findchangepts* function was used to find the three changepoints in which the mean and slope of the SCINSEV sum changes most significantly. The SCINSEV sum was transformed using MATLAB’s *fliplr* function beforehand so that *x* = 1 becomes *x* = 16 etc., leading to the lowest changepoint potentially being indicative for a change from extracochlear to the intracochlear electrode. Fourth, a polynomial fit (using *polyval* and *polyfit*) was performed on the four segments connecting the three changepoints. An example of these fitted segments is shown in Figure 3C.

The presence of extracochlear electrodes was assessed by calculating the ratio between the slope of the polynomial fit of the second (second most basal) segment and the slope of the polynomial fit of the first (most basal) segment. This is the hypothesized zone between the intracochlear and extracochlear electrodes. The sensitivity and specificity were calculated for a total of 200 different cutoff values for this slope ratio, in the range of 0–4. Receiver operating characteristic (ROC) analysis was performed to determine the most discriminative cutoff value for the ratio between the first and second segment. A correct identification was scored (a “hit”) when the presence of extracochlear electrodes, according to this cutoff points, corresponded to the presence of extracochlear electrodes at visual inspection. An incorrect identification was scored when extracochlear electrodes were identified when none were observable visually (a “false alarm”). The most discriminative cutoff value was optimized on the first four out of eight datasets, in which one dataset is equal to one cadaveric head, and tested on the other four datasets.

The quantification of extracochlear electrodes was automated by taking the most basal changepoint of the SCINCSEV sum, which was hypothesized to be in between the most basal intracochlear electrode and the most apical extracochlear electrode, and rounding that number down to the nearest integer. The quantification was marked as correct whenever this nearest integer corresponded to the number of extracochlear electrodes identified visually. The mean and its 95% confidence interval were calculated for each set with the same number of actual extracochlear electrodes.

### Patient SCINSEV Recordings

As part of intraoperative testing at our facility, SCINSEVs were recorded during insertion of the electrode array into the cochlea while there was 0.9% saline present in the middle ear. This saline is present in the middle ear due to irrigation during drilling, and because we flood the middle ear with saline to reduce pressure pulses when the round window is opened. Since we pause during insertion to slow down insertion times, trying to minimize insertion trauma to remaining hearing, SINSEVs were taken during these pauses. These telemetry measurements were collected for clinical purposes in four patients, using AB’s Volta software as described in the “SCINSEV recordings” section above, and were fully anonymized before analyses regarding extracochlear electrodes. During insertion, the number of extracochlear electrodes was noted during visual inspection through the surgical microscope by the operating surgeon, at the time of the SCINSEV recordings, but without any knowledge of the SCINSEV results. Since these measurements are part of intraoperative testing, no repeats were done and so only one measurement per patient is shown.

One CI user in our clinic was identified as having three extracochlear electrodes on CT. The contact impedances and SCINSEVs were measured as part of routine checkups. These data were fully anonymized before analyses regarding extracochlear electrodes.

## RESULTS

### Extracochlear Electrodes and SCINSEV Recordings in Human Cadaveric Heads

**Fig. 4.**
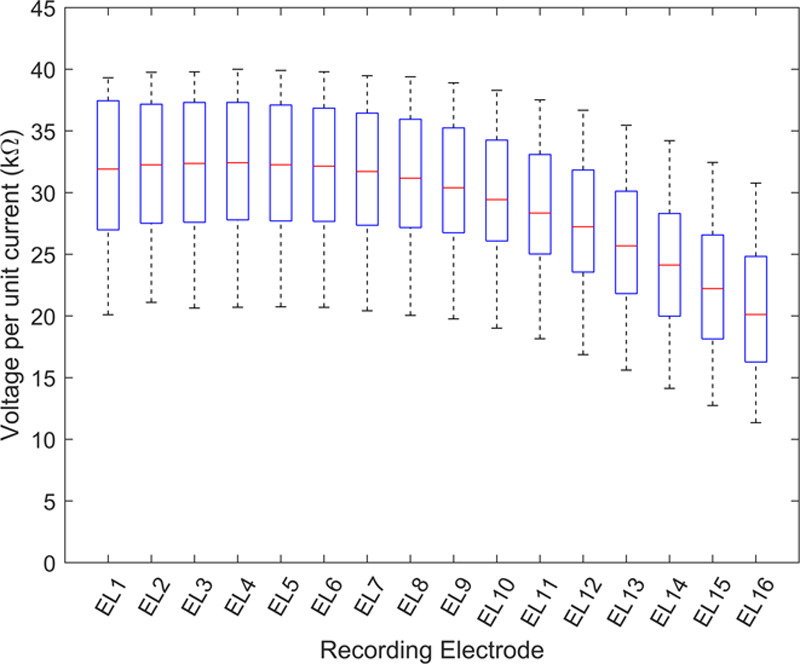
Boxplot of the values of the SCINSEV sum for full insertion in eight different heads. A variation in range of values is seen for the different specimens. Overall, a decreasing trend is seen toward the basal end of the electrode array (EL16). EL1 (electrode 1) refers to the most apical electrode, while EL16 (electrode 16) refers to the most basal electrode. Each box represents the edges of the 25th to 75th percentile, while the central mark indicates the median. No outliers are present in this dataset.

**Fig. 5.**
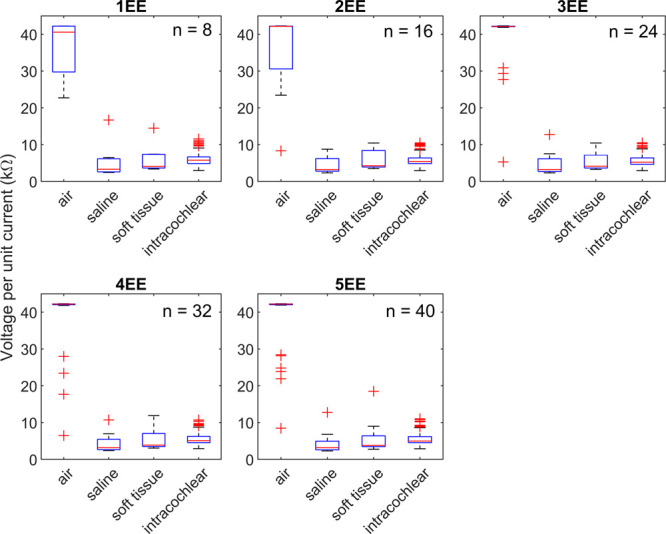
Boxplots of the contact impedances for different conditions and number of extracochlear electrodes (EE, extracochlear electrode, e.g., 2EE, 2 extracochlear electrodes). Boxplots are plotted of the measured contact impedances of the extracochlear electrodes in air, 1.0% saline and soft tissue, and these are compared with the intracochlear contact impedances for these conditions. The n number refers to the number of datapoints for each extracochlear condition. For example, 1EE has 8 datapoints for air, saline and soft tissue (8 heads), whereas for 2EE there are (2 × 8) = 16 datapoints, etc. Similarly, the number of datapoints for the intracochlear condition can be calculated as (16 − number of extracochlear electrodes) multiplied by 8 (the number of specimens). Each box represents the edges of the 25th to 75th percentile, while the central mark indicates the median. The red “+” marks represent the outliers. Note that the maximum measurable value for this implant’s software is equal to 42.358 kΩ.

**Fig. 6.**
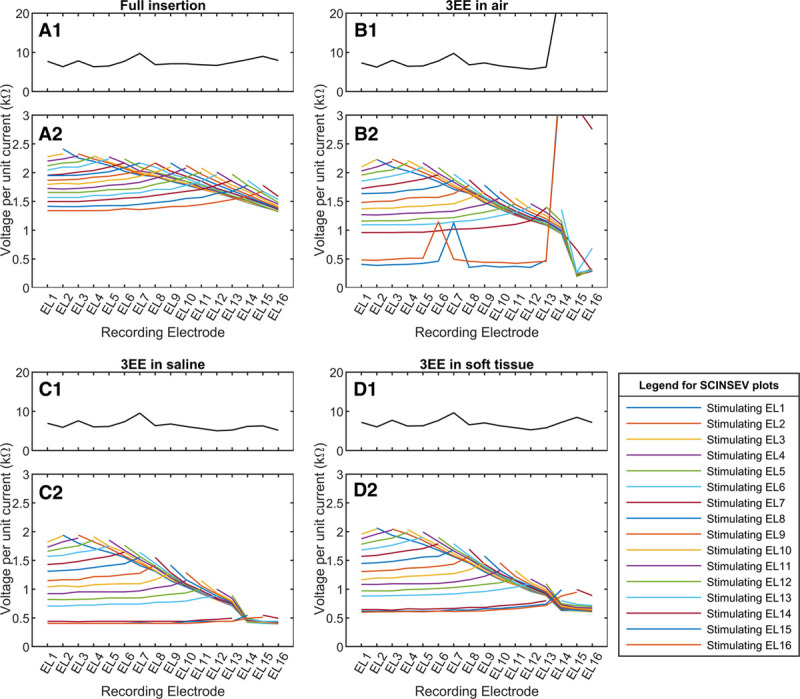
Contact impedances and SCINSEV for 1 of the specimens with 3 extracochlear electrodes. Data are shown as the mean of 3 repeats. The top graphs (e.g., A1) show the contact impedances, while the bottom graphs contain the SCINSEVs (e.g., A2). EL1 (electrode 1) refers to the most apical electrode, while EL16 (electrode 16) refers to the most basal electrode. A, Full insertion. B, Three extracochlear electrodes in air. C, Three extracochlear electrodes in 1.0% saline. D, Three extracochlear electrodes in soft tissue.

Full insertion of the CI in a flushed cochlea resulted in contact impedances in the range of 6.06 ± 2.99 kΩ (mean ±2SD). The sum of the SCINSEVs for full insertion showed a variance in range, but followed a similar trend with lower overall values toward the basal end of the cochlea (Fig. 4). Overall, contact impedances were high whenever the individual electrodes were located in air, with the majority of contact impedances reaching the maximum measurable value of 42.358 kΩ for this implant’s software. The contact impedances remained low for electrodes in saline or soft tissue and were similar to intracochlear contact impedances (Fig. 5). An example of SCINSEVs and contact impedances for an insertion with three extracochlear electrodes in one of the eight implantations is shown in Figure 6. In the case of three extracochlear electrodes, the transition from intracochlear to extracochlear is expected between electrode 13 and 14. As can be seen in Figure 6, a difference in the slope of the SCINSEV is visible at this transition zone from electrode 13 to 14 for all three conditions (Figs. 6B2, C2, D2). However, this difference is not visible in the contact impedances in the cases where the extracochlear electrodes are located in saline or soft tissue (Figs. 6C1, D1).

**Fig. 7.**
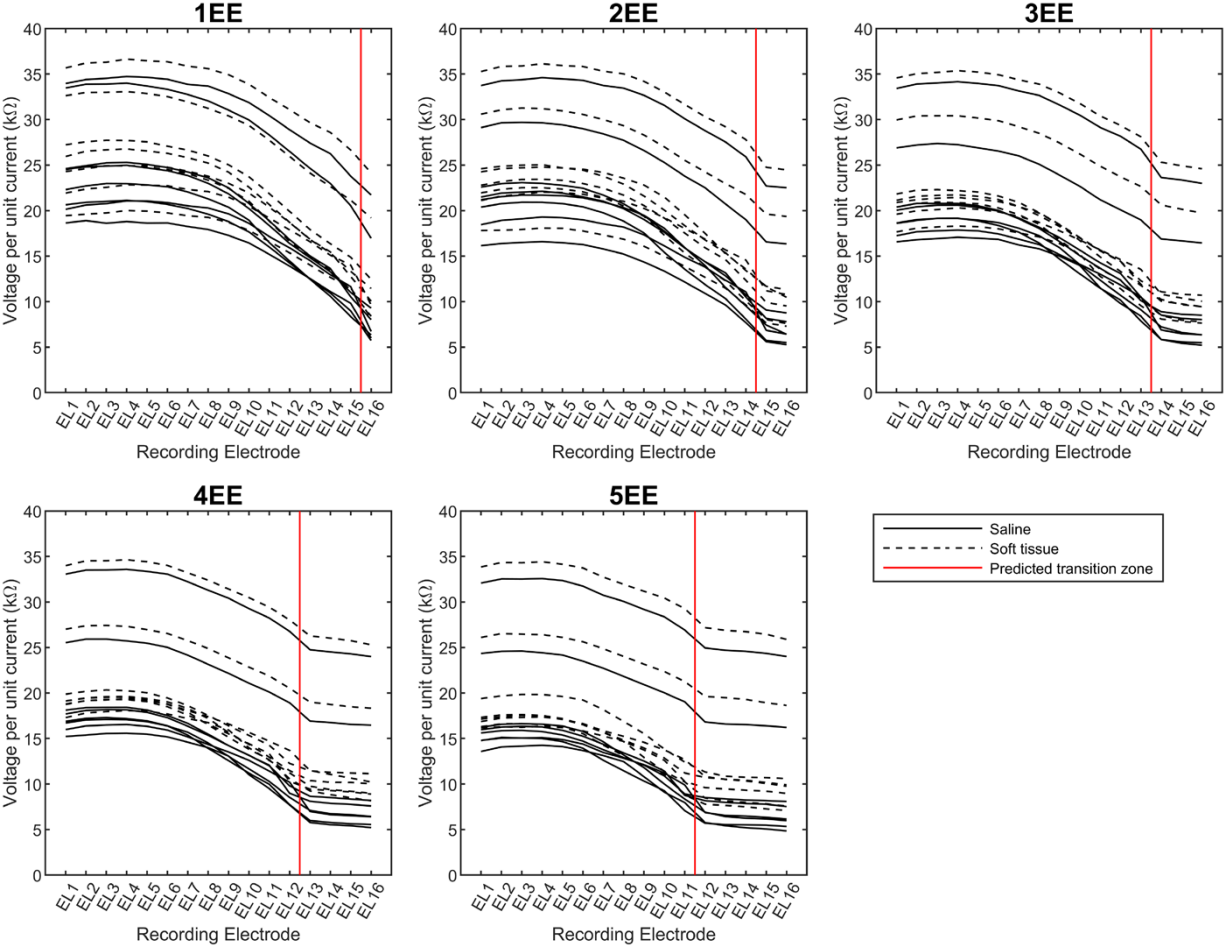
Sum of SCINSEV plotted for 1 through 5 EE (extracochlear electrodes) in saline and soft tissue. The SCINSEV sum is taken from the mean of three repeats. A vertical reference line is plotted for the predicted transition zone from intracochlear to extracochlear electrodes, corresponding to the round window opening. EL1 (electrode 1) refers to the most apical electrode, while EL16 (electrode 16) refers to the most basal electrode.

The SCINSEV sum for all specimens is shown for 1–5 extracochlear electrodes in saline and soft tissue in Figure 7. A vertical reference line is plotted for the predicted transition zone from intracochlear to extracochlear, according to visual inspection during insertion. Hypothetically, this reference line would correspond to the round window opening. Subjectively, a difference in slope is seen for the SCINSEV sum apical from the reference line compared with basally from the reference line.

**Fig. 8.**
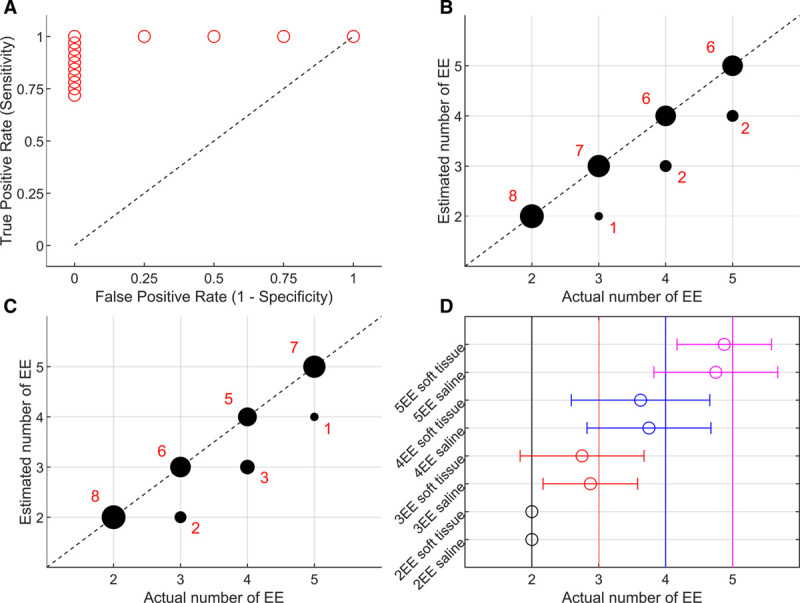
Detection and quantification of extracochlear electrodes (EE) using the automation process. A, Receiver operating characteristic (ROC, true positive rate [hit] vs. false positive rate [false alarm]) curve for detection of extracochlear electrodes. A range of 200 different cutoff points from 0 to 4 was tested for 4 out of 8 datasets. Subsequently, most discriminative cutoff value was tested on the remaining 4 out of 8 datasets. B, Predicted and actual number of extracochlear electrodes (EE) in 1.0% saline. The reference line shows the correct number of extracochlear electrodes, while the scatter plots show what was predicted. The size of the bubble corresponds to the number of specimens with that specific combination of estimated and actual number of extracochlear electrodes. The absolute number of specimens per datapoint is plotted next to the corresponding bubble. C, Predicted and actual number of extracochlear electrodes (EE) in soft tissue. The reference line shows the correct number of extracochlear electrodes, while the scatter plots show what was predicted. The size of the bubble corresponds to the number of specimens with that specific combination of estimated and actual number of extracochlear electrodes. The absolute number of specimens per datapoint is plotted next to the corresponding bubble. D, The mean and 95% confidence interval for the quantification of extracochlear electrodes. The colors of the vertical reference lines correspond to the mean and 95% confidence interval plotted for the number of extracochlear electrodes noted on the *x* axis.

The changepoints and polynomial fits of the SCINSEV sum were calculated for full insertion and 1–5 extracochlear electrodes in saline and soft tissue. This method proved not to be effective for SCINSEVs with one extracochlear electrode and was therefore excluded from further optimization of the detection tool. The ratio of the slope of the most basal intracochlear segment and extracochlear segment for 2–5 extracochlear electrodes was found to be in the range of 2.12 to 22.15 for saline and 1.73 to 17.28 for soft tissue. For a full insertion, the ratio of the two segments surrounding the most basal changepoint was in a range of 0.33–1.09. A range of 200 cutoff points (values 0–4 with step size 0.02) was tested for best identification of extracochlear electrodes in the first four out of eight datasets. The ROC curve is plotted in Figure 8A. A maximum sensitivity of 100.0% and specificity of 100.0% was reached for 2–5 extracochlear electrodes with cutoff values ranging from 0.74 to 2.32. The median value in this range, 1.53, was tested on the remaining four datasets. When the ratio was below 1.53, the SCINSEV was marked as a full insertion, while above 1.53 was marked as presence of extracochlear electrodes. This was compared with the presence of extracochlear electrodes at visual inspection through the surgical microscope. Specificity and sensitivity were both 100% for these remaining datasets.

The exact quantification of extracochlear electrodes was tested for detection of 2–5 extracochlear electrodes in saline and soft tissue, that is, if the exact number of extracochlear electrodes was identified. For example, if four electrodes were extracochlear, 3 or 5 electrodes identified as extracochlear would be considered incorrect. Eighty-four percent (27 out of 32) of the extracochlear electrodes in saline were correctly quantified, while 81% (26 out of 32) were correctly quantified for soft tissue. The predicted and actual number of extracochlear electrodes is plotted in Figures 8B, C. In all cases, when the number of predicted extracochlear electrodes diverged from the actual number, the estimated number diverged by −1. In other words, the automated detection tool might underestimate the number of extracochlear electrodes by 1. The mean of the estimated number of extracochlear electrodes and its 95% confidence interval is shown in Figure 8D.

**Fig. 9.**
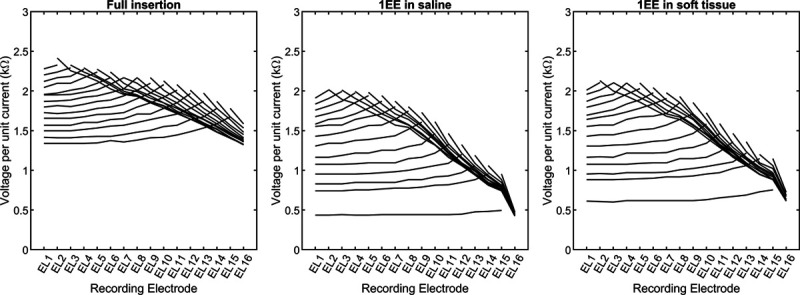
Example of 1 extracochlear electrode (EE) in 1.0% saline and soft tissue. SCINSEVs are shown as the mean of 3 repeats. The SCINSEV for a full insertion measured in the same specimen is shown as a reference. EL1 (electrode 1) refers to the most apical electrode, while EL16 (electrode 16) refers to the most basal electrode.

The analysis tool for extracochlear electrodes was not sufficiently sensitive for 1 extracochlear electrode, even though the SCINSEVs themselves may show a recognizably different pattern on visual inspection. Figure 9 shows a SCINSEV for full insertion and one extracochlear electrode in saline or soft tissue, measured in the same specimen. The longitudinal spread toward the apex shows a different pattern for the extracochlear electrode 16 in both conditions, when compared with the full-insertion SCINSEV measurement.

### Intraoperative SCINSEV Recordings During Slow Insertion

**Fig. 10.**
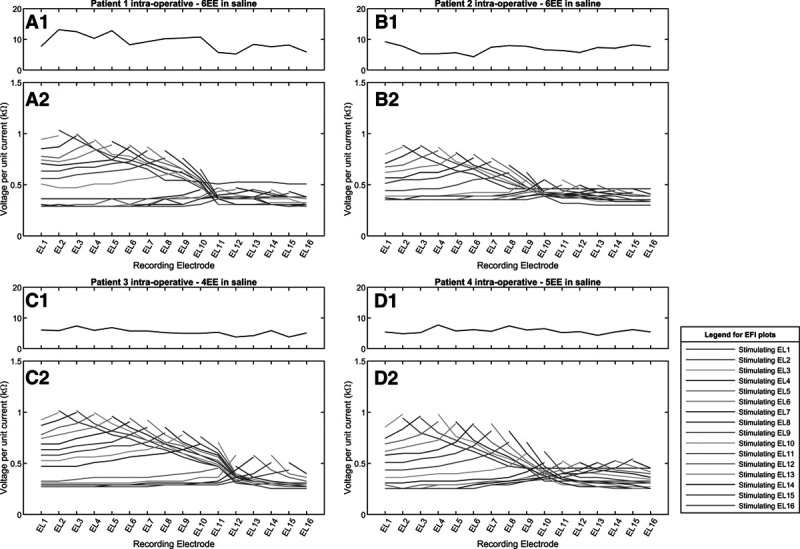
Contact impedances and SCINSEVs for four intraoperative patients with extracochlear electrodes. These intraoperative recordings were made during slow insertion of the electrode. The extracochlear electrodes were surrounded by 0.9% saline during insertion. The top graphs (A1, B1, C1, D1) show the contact impedances, while the bottom graphs contain the SCINSEVs (A2, B2, C2, D2). EL1 (electrode 1) refers to the most apical electrode, while EL16 (electrode 16) refers to the most basal electrode. A, Six extracochlear electrodes for patient 1, measured intraoperatively in 0.9% saline. B, Six extracochlear electrodes for patient 2, measured intraoperatively in 0.9% saline. C, Four extracochlear electrodes for patient 3, measured intraoperatively in 0.9% saline. D, Five extracochlear electrodes for patient 4, measured intraoperatively in 0.9% saline.

To test for actual clinical utility, as part of intraoperative testing, SCINSEVs were recorded during insertion with the Advanced Bionics HiRes Ultra with a HiFocus SlimJ electrode. Intraoperative SCINSEVs and contact impedance for four patients are shown in Figure 10. During surgery, 0.9% saline is present in the middle ear surrounding the extracochlear electrodes. For two out of four intraoperative recordings shown, six electrodes were located extracochlearly (Figs. 10A, B), while for one of the patients (Fig. 10C) four electrodes and for another (Fig. 10D) five electrodes were located extracochlearly. The analysis tool was tested on these patient’s SCINSEVs. For the intraoperative patient SCINSEVs with six and five extracochlear electrodes, the ratio of the most basal intracochlear segment to the extracochlear segment was higher than the previously described cutoff of 1.53 (3.234 for patient 1, 2.909 for patient 2, and 4.5676 for patient 4) and therefore the tool identified extracochlear electrodes correctly on these SCINSEVs. The quantification of extracochlear electrodes was determined at six extracochlear electrodes for patient 1, seven extracochlear electrodes for patient 2, and five extracochlear electrodes for patient 4. For the intraoperative patient SCINSEV with four electrodes located extracochlearly, the ratio of the intracochlear to the extracochlear segment was 1.428 and therefore lower than the previously described cutoff of 1.53, but within the range identified as having a maximum specificity and sensitivity for the cadaver experiments (0.74–2.32). The quantification of extracochlear electrodes in this patient was determined as 5, while only four electrodes were located outside of the cochlea as established by inspection through the surgical microscope. Naturally, as these measurements were taken during slow insertion of the implant, the electrode array was fully inserted after these measurements.

### Postoperative SCINSEV Recordings in Patient with Known Extracochlear Electrodes

**Fig. 11.**
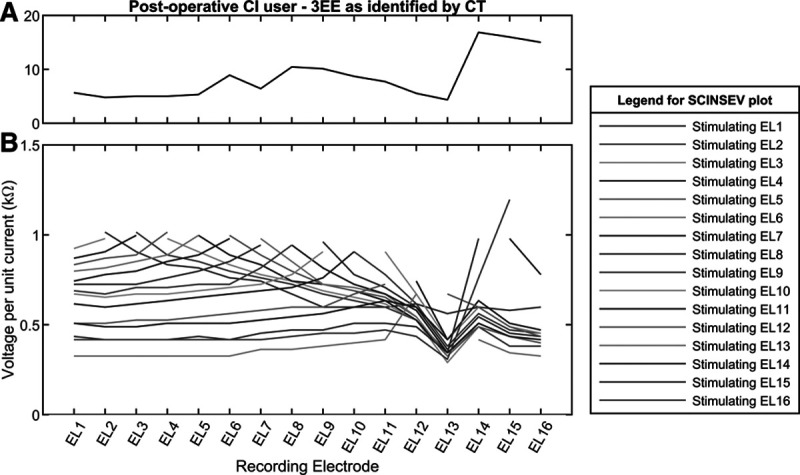
Contact Impedances and SCINSEV for a postoperative CI user with three known extracochlear electrodes as identified by CT scanning. The top graph shows the contact impedances (A) while the bottom graph shows the SCINSEV (B). EL1 (electrode 1) refers to the most apical electrode, while EL16 (electrode 16) refers to the most basal electrode.

One CI user in our clinic, implanted with the Advanced Bionics HiRes Ultra with a HiFocus SlimJ electrode, was identified to have three extracochlear electrodes by postoperative CT scanning. The anonymized SCINSEV data (Fig. 11) were analyzed and showed a ratio of the intracochlear to the extracochlear segment of 4.773 (above the cutoff of 1.53), while the quantification correctly showed three electrodes to be extracochlear. In this example, the contact impedances on the extracochlear electrodes (15.009–16.858) were already elevated when compared with the intracochlear electrodes (4.350–10.441), likely indicating that these electrodes were surrounded by air.

## DISCUSSION

This study shows that SCINSEVs have good potential as a detection tool for extracochlear electrodes. Even when contact impedances on extracochlear electrodes are similar to intracochlear electrodes, SCINSEVs showed a change in the spread of current-induced voltage from intracochlear to extracochlear electrodes. This is the case for both extracochlear electrodes in saline, simulating intraoperative conditions and directly postoperative conditions, and when soft tissue is wrapped around the extracochlear electrodes, simulating fibrotic tissue formation and therefore long-term postoperative conditions. The contact impedances were only useful in identifying extracochlear electrodes if the extracochlear electrodes were in air. This is consistent with the study by Holder et al. (2018), showing that a substantial subset of live CI users with extracochlear electrodes, as clinically identified by CT scanning, had normal contact impedances. Furthermore, normal contact impedances were seen on the extracochlear electrodes in the four intraoperative SCINSEV recordings in live patients during insertion (Fig. 10).

The automation process shows high sensitivity and specificity for the detection of extracochlear electrodes in cadavers, and seems promising in preliminary live human data. For clinicians unfamiliar with SCINSEVs, this tool could be highly beneficial for initial detection of extracochlear electrodes. Since most migration of electrodes is expected during the final stages of CI surgery, (van der Marel et al. 2012) using this detection tool during this time might prevent re-insertion surgery for some CI users (Brown et al. 2009; Connell et al. 2008; Green et al. 2004; Wang et al. 2014). For clinicians familiar with SCINSEVs, visual inspection of the SCINSEV pattern could be enough to quickly recognize the change in pattern between intracochlear and extracochlear electrodes. In general, a clear transition zone in the SCINSEV pattern is seen between intracochlear and extracochlear electrodes with a collapse of the SCINSEV in the extracochlear region.

Some limitations of this study exist. For one extracochlear electrode, the automated analysis tool proved unsuccessful in detecting and quantifying the extracochlear electrodes. In all cases, this was due to the *findchangepts* MATLAB (vR2018A, MathWorks Inc., MA) function not correctly identifying the most basal changepoint and therefore this basal segment with one extracochlear electrode had less influence on the mean and slope of the polynomial fit of the SCINSEV sum than with two or more extracochlear electrodes. Although the quantification of one extracochlear electrode was ineffective, a difference in SCINSEV compared with baseline is still seen by visual inspection and could assist the clinician experienced with SCINSEVs in quantifying the extracochlear electrodes.

Implantation in fresh-frozen human cadaveric heads provides the opportunity to systematically test multiple conditions without variation in anatomy between these conditions. However, some limitations of this method do exist. First, we are unaware of the condition of the cochlea after the freezing and defrosting process. Second, flushing the cochlea through the lateral semicircular canal might destroy the fragile structures inside the cochlea and therefore change the environment of the electrode array. However, the SCINSEV shapes from cadaveric cochleae are similar to those we see in living subjects, although the range of detected voltages is different (an example is shown in Fig. 2). Furthermore, intraoperative recordings in live patients showed that a similar SCINSEV pattern is seen for extracochlear versus intracochlear electrodes during insertion (Fig. 10). Another limitation is that the soft tissue placed on the extracochlear electrodes is fresh and therefore has not yet scarred into place, which may change its long-term in vivo characteristics in real life. It should also be noted that in case of air bubbles or broken electrodes at the basal end of the cochlea, these might limit the ability of this tool to detect the transition from intracochlear to extracochlear electrodes.

Another possible limitation of this study includes the use of SCINSEVs that show little variation in shape between cadavers. Differences in SCINSEV shape is reported in case of tip fold-over and mid-cochlear shunts (Vanpoucke et al. 2004; Zuniga et al. 2017). Although the SCINSEV sum uses all datapoints, and is therefore not expected to be very susceptible to outliers, an overall change of SCINSEV shape might change the ability of the automatized detection tool to correctly detect and quantify extracochlear electrodes. Additional analysis should be done to study the effect of SCINSEV shape on the detection rate of extracochlear electrodes in living CI users. Furthermore, while the most discriminative cutoff range of 0.74–2.32 used 32 SCINSEVs (four specimens with eight different extracochlear conditions) to determine the upper limit, only four different full insertion SCINSEVs from the four specimens were available to determine the lower limit of this range. However, the range of most discriminative cutoffs (0.74–2.32) in cadavers seems to translate to intraoperative SCINSEV recordings in patients (Fig. 10) and the one postoperative case shown (Fig. 11). Nonetheless, validation and optimization of this range on a larger cohort of intraoperative and postoperative CI users will be necessary. A general method is shown, and it is possible that the exact parameter values for the fitting algorithms to detect extracochlear electrodes may be different for different electrode types and postoperative versus intraoperative use. However, our initial live CI user data seems to support its ability to detect extracochlear electrodes in living subjects.

In summary, SCINSEVs show good potential for detection of extracochlear electrodes, even when contact impedances remain consistent with intracochlear contact impedances, and could potentially replace CT scans for the initial screening for extracochlear electrodes in the long run. Although proof of concept is shown in five live CI users, larger studies are necessary for the validation of this concept. The automated tool could potentially assist clinicians in detecting and quantifying the extracochlear electrodes as it is developed in time. The quantification of extracochlear electrodes with the automated tool could potentially benefit clinical mapping. Furthermore, it might assist in troubleshooting non-auditory sensations for some CI users. Further studies will include validation in live CI users for both intraoperative and postoperative conditions, improving detection and quantification in case of one extracochlear electrode, as well as further analysis in case of differences in SCINSEV shape (for example as suspected in case of cochlear malformations, broken electrodes or tip fold-over) and exploration of this tool with other types of electrodes and CI manufacturers.

## ACKNOWLEDGMENTS

The authors would like to thank Alan Archer-Boyd and François Guérit for their helpful notes on this project, Patrick Boyle from Advanced Bionics UK for his help in providing cochlear implants, and Robert MacFarlane for his help in the Microsurgical Skills Lab.
